# STS-1 and STS-2, Multi-Enzyme Proteins Equipped to Mediate Protein–Protein Interactions

**DOI:** 10.3390/ijms24119214

**Published:** 2023-05-24

**Authors:** Barbara Hayes, Peter van der Geer

**Affiliations:** Department of Chemistry and Biochemistry, San Diego State University, 5500 Campanile Dr., San Diego, CA 92105, USA; bhayes7750@sdsu.edu

**Keywords:** STS-1, STS-2, protein–tyrosine kinases, BCR-ABL1, Cbl, EGF receptor, SH3

## Abstract

STS-1 and STS-2 form a small family of proteins that are involved in the regulation of signal transduction by protein–tyrosine kinases. Both proteins are composed of a UBA domain, an esterase domain, an SH3 domain, and a PGM domain. They use their UBA and SH3 domains to modify or rearrange protein–protein interactions and their PGM domain to catalyze protein–tyrosine dephosphorylation. In this manuscript, we discuss the various proteins that have been found to interact with STS-1 or STS-2 and describe the experiments used to uncover their interactions.

## 1. Discovery of STS Family Proteins

Scott and co-workers were characterizing RNA transcripts that originate in an area of chromosome 21 that is linked to a familial form of deafness. They isolated a full-length transcript that encodes a protein with a UBA (ubiquitin-associated) domain and an SH3 (Src-homology 3) domain and named it UBASH3A [1]. Aravind and coworkers used sensitive computational sequence analysis tools to define a new superfamily of phosphoesterases [2]. While members of this superfamily have poorly conserved amino acid sequences, they all contain two conserved histidine residues that are essential for catalysis. UBASH3 proteins were found to be part of this superfamily [2]. Their phosphoesterase domain is present between the UBA domain and the SH3 domain [2]. JAK2 is a cytoplasmic protein–tyrosine kinase that is involved in cytokine receptor signaling [3]. Ihle and coworkers isolated a 70 kDa protein by affinity chromatography, using a phosphopeptide based on tyrosine 966 in JAK2. They described p70 as a protein with an SH3 domain and a carboxy-terminal phosphoglycerate mutase domain [4]. Cbl is a negative regulator of receptor protein–tyrosine kinase signaling [5,6]. Tsygankov and coworkers identified TULA (T cell Ubiquitin LigAnd), as a protein they copurified with Cbl, while Dikic and coworkers identified Clip4 in a two-hybrid screen with Cbl [7,8]. In this manuscript we will refer to p70, UBASH3B and TULA-2 as suppressors of T cell signaling 1 or STS-1; we will refer to UBASH3A, TULA-1, and Clip-4 as STS-2.

## 2. STS-1 and STS-2 an Introduction

**Figure 1:**
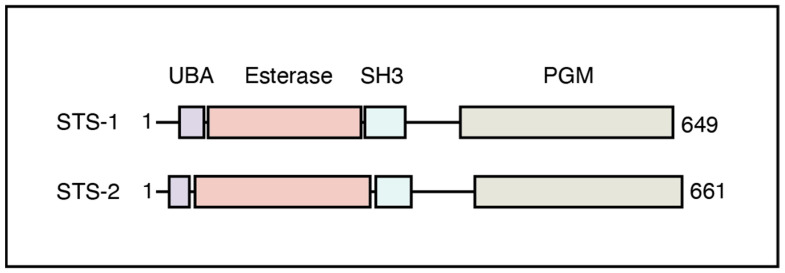
Schematic representation of STS-1 and STS-2.

STS-1 and STS-2 form a small family of soluble proteins that are present in the cytoplasm of many cell types. They are composed of an amino-terminal UBA domain, followed by a phosphodiesterase domain, an SH3 domain, and a carboxy-terminal PGM (phosphoglycerate mutase) domain (Figure 1). UBA domains mediate protein–protein interactions by binding to ubiquitylated proteins [9]. Phosphoesterase domains hydrolyze phosphoesters in a variety of substrates [2]. The STS-1 and STS-2 esterase domains remain poorly characterized. SH3 domains also mediate protein–protein interactions. They do this by binding to Pro-X-X-Pro sequences in other proteins [10]. The PGM domains are related to phosphoglycerate mutases. Phosphoglycerate mutases have similarities in structure and catalytic mechanism with protein phosphatases [11]. This realization directed further studies into the function of the PGM domain. It was found that the isolated STS-1 PGM domain, expressed in E. coli, dephosphorylates phosphotyrosine-containing peptides [12,13]. In addition, the STS-1 PGM domain dephosphorylates several different proteins in vitro, while expression of the full-length STS-1 protein in mammalian cell lines resulted in dephosphorylation of phosphotyrosine-containing proteins [12,13]. This protein-phosphatase activity was dependent on the presence of two conserved histidine residues within the PGM domain [12]. No activity towards phosphoserine- or phosphothreonine-containing peptides was detected [12]. Interestingly, the STS-2 PGM domain has no or very little protein–tyrosine phosphatase activity [12,14]. Thus, STS-1 and STS-2 are multifunctional enzymes that are equipped with a UBA and an SH3 domain to mediate interactions with other proteins.

## 3. JAK2

Janus kinases are protein–tyrosine kinases that are associated with various cytokine receptors, including interleukin receptors, the GM-CSF receptor, and the EPO receptor [15]. JAK2, a member of the Janus kinase family, is composed of an amino-terminal FERM (four-point-one, ezrin, radixin, moesin) domain, followed by an SH2-like domain, a pseudokinase domain, and a protein–tyrosine kinase domain [16]. The FERM domain and the SH2-like (Src-homology 2-like) domain mediate association with the receptor [16]. The pseudo-kinase domain regulates the protein–tyrosine kinase domain [16]. Ligand binding to the receptor causes activation of the receptor-associated JAK2 kinase. This involves phosphorylation on tyrosine residues within the activation loop. Following activation, the kinase phosphorylates additional tyrosine residues within its own polypeptide chain, as well as tyrosine residues within the receptor [15,16]. Tyrosine phosphorylation sites in the receptor were already known to act as binding sites for STAT transcription factors and other signaling proteins [15,16].

To investigate whether tyrosine phosphorylation sites on JAK2 can also function as binding sites for signaling proteins, Ihle and coworkers linked a synthetic phosphopeptide identical to a major JAK2 autophosphorylation site, Tyr (tyrosine) 966, to Sepharose beads [4]. These phosphopeptide beads were incubated with cell lysates and bound proteins were analyzed by SDS-PAGE, followed by immunoblotting. It was found that the P.Tyr (phosphotyrosine) 966 peptide bound several known signaling proteins, including ShcA, PI-3 kinase, and an unidentified 70 kDa protein [4]. Following large-scale purification, protease digestion, and peptide sequencing, p70 was identified as a protein with an SH3 domain and a phosphoglycerate mutase domain. This protein is now known as STS-1 [4]. These observations suggested that the STS-1 protein forms a complex with JAK2 within the cell. This conclusion was confirmed by probing JAK2 immunoprecipitations for the presence of Flag-tagged STS-1 and vice versa [4]. It remains unclear exactly which part of STS-1 is involved in the interaction with JAK2 and whether the interaction between JAK2 and STS-1 is direct or indirect. Because STS-1 does not contain a domain known to bind stably to phosphotyrosine, the authors suggested in their manuscript that the interaction between STS-1 and JAK2 could be indirect.

## 4. ZAP-70

ZAP-70 is a cytoplasmic protein–tyrosine kinase that is composed of two amino-terminal SH2 domains and a carboxy-terminal protein–tyrosine kinase domain. The SH2 domains mediate phosphorylation-dependent protein–protein interactions by binding to phosphorylated tyrosine residues in its binding partners [17]. ZAP-70 is involved in signal transduction by the T cell receptor [18]. Its cousin Syk is involved in signal transduction by the B cell receptor [18]. T cell receptor activation involves the recruitment of an Src-family protein–tyrosine kinase that is linked to a co-receptor to the T cell receptor complex. The Src-family protein–tyrosine kinase phosphorylates the ζ chain of the receptor complex on a set of tyrosine residues that act as binding sites for the two ZAP-70 SH2 domains [18]. Following binding to the T cell receptor complex, ZAP-70 is activated and phosphorylates additional signaling proteins, including LAT and SLP76, that are instrumental in activating the signal transduction cascades that result in T cell activation [18,19].

Gene knockout experiments carried out by Ihle and coworkers led to the observation that mice lacking functional genes for both STS-1 and STS-2 have hyperactive T cells [20,21]. T-cell receptor crosslinking resulted in normal activation of the Src-like kinases that phosphorylate the receptor. Further analysis showed an increase in tyrosine phosphorylation of ZAP-70, which is activated downstream of the receptor [18,20]. The ZAP-70 substrates LAT and SLP76 also showed increased levels of tyrosine phosphorylation. These studies suggest that STS-1 and STS-2 function as negative regulators of signal transduction by the T cell receptor. They appear to act by decreasing ZAP-70 tyrosine phosphorylation as well as by decreasing its kinase activity.

In contrast to what was observed in gene knockout studies, overexpression studies in Jurkat cells suggested that STS-2 increases T-cell receptor signaling [7]. In addition, STS-2 blocks the decrease in T cell receptor signaling that is caused by Cbl overexpression [7]. Reduction of STS-2 expression in Jurkat cells, using RNAi, resulted in reduced T cell receptor signaling and decreased ZAP-70 tyrosine phosphorylation following T cell receptor stimulation [7]. Thus, work in T cell receptor-expressing cell lines suggest that STS-2 is an activator of T cell receptor signaling.

These observations prompted Tsygankov and coworkers to investigate whether STS-1 and STS-2 proteins also regulate the Syk protein–tyrosine kinase. To do so, they overexpressed STS-1 or STS-2, together with Syk and a chimeric receptor composed of the extracellular domain and transmembrane region of CD-8 and the cytoplasmic region of the T cell receptor ζ chain [22]. Their experiments showed the coimmunoprecipitation of both STS-1 and STS-2 with Syk [22]. STS-1 was shown to reduce Syk tyrosine phosphorylation. In contrast, STS-2 was shown to increase Syk tyrosine phosphorylation. The investigators concluded that STS-1 uses its phosphoglycerate mutase domain to dephosphorylate Syk. Overexpression of STS-2, on the other hand, most likely interfered with the recruitment of STS-1, thereby inhibiting the dephosphorylation of Syk, possibly resulting in its activation. It remains unclear how STS-1 or STS-2 associate with ZAP-70 or Syk.

## 5. CIN85

CIN85 was identified as an 85 kDa protein that associates with Cbl [23,24]. It is composed of 3 SH3 domains and a proline-rich region. Isoforms that lack the first or the first two SH3 domains can be generated through alternative splicing. CIN85 proteins are involved in signal transduction, receptor endocytoses, and cytoskeletal remodeling. They are thought to be involved in the internalization of receptor protein–tyrosine kinases by recruiting Cbl, endophilin, and dynamin [23,24].

Interestingly, a T cell-specific CIN85 knock-out mouse has a phenotype that is very similar to the STS-1 and STS-2 double knockout [25]. T cells are hyperactive and show increases in IL-2 secretion and elevated levels of ZAP-70 and SLP76 tyrosine phosphorylation [25]. This phenotype can be rescued by the expression of wild-type CIN85, but not by expression of CIN85 mutants lacking one or more SH3 domains or the proline-rich region.

To identify CIN85 bound proteins involved in inhibition of T cell receptor signaling wild type CIN85 and a mutant lacking the proline-rich region were isolated by immunoprecipitation. CIN85 and associated proteins were digested with trypsin and peptides were identified by HPLC and mass spectrometry. This led to the identification of STS-2 as a CIN85-associated protein [25]. The interaction between CIN85, Cbl, and STS-2 was confirmed by immunoprecipitation and was found to increase upon T cell receptor stimulation. Association with STS-2 was dependent on the presence of the CIN85 SH3 domains and the proline-rich region. It is likely that the binding of the STS-2 SH3 domain to the CIN85 proline-rich region plays a role in the association. No binding of STS-1 to CIN85 was observed.

## 6. Cbl

Cbl proteins are E3 ubiquitin ligases that negatively regulate signaling by protein–tyrosine kinases [5,6]. Cbl proteins are composed of a tyrosine-kinase binding domain, a ring finger domain, a proline-rich region, and a less conserved carboxy terminus. The tyrosine-kinase binding domain binds to tyrosine phosphorylation sites in Cbl targets and the ring finger domain binds E2 ubiquitin-conjugating enzymes that transfer activated ubiquitin to target proteins [5,6]. The attachment of ubiquitin to membrane proteins helps initiate protein trafficking and degradation [26]. The proline-rich region provides docking sites for SH3 domain-containing proteins that assist Cbl in targeting proteins for internalization and degradation [5,6]. Tyrosine phosphorylation sites on Cbl proteins can act as binding sites for SH2 domain-containing signaling proteins including, PI-3 kinase, Crk-I, and Vav [5,6].

To learn more about the biochemistry of Cbl, Tsygankov, and coworkers purified Cbl and associated proteins from a human T cell line. They took advantage of the fact that the human Cbl protein contains a string of seven histidine residues close to its amino-terminal end that can bind to a Ni-affinity column. Proteins eluted from the Ni-affinity column were further purified using an anti-Cbl affinity column. Bound proteins were resolved by SDS-PAGE, digested with trypsin, and identified by mass spectroscopy [7]. This led to the identification of several Cbl-associated proteins, including CIN85, PI-3 kinase, and STS-2 [7]. At approximately the same time, Dikic and coworkers carried out a two-hybrid screen, in which they identified several Cbl-interacting proteins that included Clip-4 or STS-2 [8].

Co-immunoprecipitation experiments in which both proteins were overexpressed showed that Cbl and STS-2 interact within the cell. Deletion of the proline-rich region in Cbl or mutation of the SH3 domain in STS-2 blocked co-immunoprecipitation [7,8]. Purified GST-STS-2 fusion proteins expressed in E. coli bound to Cbl present in cell lysates of 293 cells and binding was found to be dependent on the presence of a functional SH3 domain on STS-2 and the Pro-rich region on Cbl [7]. The interactions between Cbl and STS-2 appeared to be independent of stimulation [8]. Together, these observations strongly suggest that the STS-2 SH3 domain mediates the interaction with Cbl by binding to the Cbl proline-rich region. However, data that show a direct interaction between these two proteins were not included.

It is well established that Cbl uses its TKB domain to bind to activated receptors [27]. Following binding, Cbl will target those receptors for internalization and degradation [5,6]. The effect of STS-2 on Cbl-dependent receptor internalization and degradation was investigated using various receptors. As anticipated, overexpression of Cbl increased the rate at which EGF receptors were removed from the cell surface [8]. Co-expression of STS-2 reduced the rate of Cbl-induced EGF receptor internalization and degradation [7,8]. This effect was dependent on the presence of functional SH3 and UBA domains on STS-2 [8]. Similarly, STS-2 expression appeared to increase PDGF receptor levels in 293T cells the in presence of Cbl, both in the absence and in the presence of PDGF [8]. These observations suggest that STS-2 reduces the ability of Cbl to remove activated receptors from the cell surface. Interestingly, overexpression of STS-2 appeared to reduce Cbl protein levels in the cell [7]. Furthermore, mutation of the STS-2 SH3 domain appeared to increase STS-2 protein levels in cells overexpressing Cbl [8]. This suggests the possibility that the formation of a complex between STS-2 and Cbl leads to its degradation.

After the identification of STS-2 as a binding partner for Cbl and a negative regulator of EGF receptor downregulation, Dikic, and coworkers looked in detail at STS-1. They observed robust binding of STS-1 to the EGF receptor. Binding was dependent on receptor activation [28]. STS-1 overexpression strongly reduced phosphotyrosine levels in the activated receptor and blocked EGF receptor downregulation [28]. Both, the reduction in receptor tyrosine phosphorylation and inhibition of receptor degradation, were dependent on the presence of a functional PGM domain and independent of the presence of a functional UBA domain [28]. These results suggest that STS-1 is recruited to the EGF receptor where it dephosphorylates the receptor, thereby reducing signal transduction as well as the recruitment of proteins involved in EGF receptor downregulation.

The binding of STS-1 to Cbl was independent of EGF receptor activation, while the binding of STS-1 to the receptor was stimulated by EGF [28]. Interestingly, overexpression of STS-1 reduced the EGF-dependent binding of Cbl to the receptor, while it increased STS-1 binding to the receptor [28]. This suggests binding of STS-1 and Cbl to the activated EGF receptor are independent process. These observations point to the idea that STS-1 may be engaged in multiple independent protein–protein interactions with separate activities.

## 7. BCR-ABL1

BCR-ABL1 is an oncogene that is formed in a chromosomal translocation between chromosome 9 and chromosome 22. This translocation involves both the BCR and the ABL1 genes and results in the formation of the Philadelphia chromosome [29]. Formation of the Philadelphia chromosome and expression of the BCR-ABL1 oncoprotein is the hallmark of chronic myelogenous leukemia [30]. BCR-ABL was also the first oncoprotein that was targeted successfully with a small molecule protein–tyrosine kinase inhibitor [31].

BCR is a multidomain protein that is widely expressed. It is composed of an amino-terminal dimerization domain, followed by a serine/threonine-protein kinase domain, a pair of SH2 domains, a Rho guanine nucleotide exchange domain, a RAC-GTPase activating domain, and a carboxy-terminal PDZ (postsynaptic density-95, disks-large, zonula occludens-1) domain [32]. The Rho guanine nucleotide exchange domain functions as an activator of the Rho family of small GTP-binding proteins [33]. The RAC GTPase activating domain helps RAC GTP-binding proteins turn themselves off [33]. ABL1 is a protein–tyrosine kinase [34]. It is composed of an amino-terminal SH3 domain, an SH2 domain, a protein–tyrosine kinase domain, and a carboxy-terminal region containing an actin-binding domain [34]. The ABL1 protein is involved in signal transduction by a wide variety of extracellular signals.

BCR-ABL1 protein is a constitutively active protein–tyrosine kinase composed of the amino-terminal half of BCR fused to most of the ABL1 protein. Depending on the location of the breakpoint in the BCR gene, the BCR-ABL1 gene produces a 190 kDa protein (p190^BCR-ABL^) or a 210 kDa protein (p210^BCR-ABL^). p210^BCR-ABL^ includes the Rho-GEF domain that is missing in p190^BCR-ABL^. Chronic myelogenous leukemia is usually associated with the expression of p210^BCR-ABL^ [35]. A fraction of acute lymphoblastic leukemias also contain the Philadelphia chromosome [35]. However, most Philadelphia chromosome-positive lymphoblastic leukemias express p190^BCR-ABL^ [35]. To better understand BCR-ABL1-dependent oncogenesis, several groups have used a proteomics approach to describe and compare the protein–protein interactions that result from the expression of either p190^BCR-ABL^ or p210^BCR-ABL^.

Superti-Furga and co-workers used an anti-ABL monoclonal linked to Sepharose beads to purify the p210^BCR-ABL^ from the K562 cells. K562 is a chronic myelogenous leukemia cell line [36]. Purified proteins were resolved by SDS-PAGE and identified by mass spectrometry. This experiment led to the identification of 18 proteins including BCR-ABL1, Cbl, STS-1, ShcA, and Grb2 [37]. To further explore the nature of the interactions between these proteins, p210^BCR-ABL^-interacting proteins were each fused to tandem affinity tags, expressed in K562 cells, and purified by two sequential rounds of affinity purification [38]. Purified protein complexes were again analyzed by SDS-PAGE and mass-spectrometry. Amino-terminally tagged STS-1, expressed in K562 cells, co-purified with 8 other proteins that included p210^BCR-ABL^, Crk-I, and SHIP2, but neither ShcA nor Cbl [37]. Amino-terminally tagged Cbl co-purified with 11 other proteins, including p210^BCR-ABL^, CRK-I, SHIP2, and ShcA [37]. Amino-terminally tagged ShcA co-purified with 27 other proteins, including p210^BCR-ABL^, CRK-I, SHIP2, STS-1, Grb2, and several adaptins [37]. Adaptins are proteins that link cargo proteins to clathrin during the formation of transport vesicles [39]. Thus, STS-1 appears to be associated with the oncogenic BCR-ABL1 protein–tyrosine kinase, Cbl, ShcA, and several other proteins. The ability to bind to other proteins in this analysis may have been affected by the location of the affinity tags.

BirA is a biotin-ligase that attaches biotin to other proteins [40]. When fused to a protein of interest and expressed in mammalian cells, BirA will attach biotin to proteins that are present in close proximity to the protein of interest [41]. Following cell lysis and digestion of cellular proteins with trypsin, biotinylated peptides can be isolated and identified by mass spectrometry [41]. Pandey and coworkers employed this approach to identify proteins that are present near STS-1 in p210^BCR-ABL^-expressing cells [42]. A mutant version of STS-1 in which the amino-terminal half of the protein, including the UBA and SH3 domain, had been deleted was used as a negative control. They identified 42 proteins that were present specifically adjacent to the full-length protein [42]. These include BCR-ABL1, Cbl, Crk-I, SHIP2, CIN85 and ShcA. Crk-I is a small adaptor protein composed of an SH2 and an SH3 domain. It plays a role in the regulation of migration and adhesion by bringing effector proteins together [43]. SHIP2 is an SH2 domain-containing phosphatidylinositol-5-phosphatase. SHIP2 is thought to play a role in the regulation of adhesion, endocytosis, and cell proliferation [44]. Based on the extent of biotinylation, it appears that ShcA may be the most proximal neighbor to STS-1 in p210^BCR-ABL^-expressing cells [42].

To compare and quantify the relative abundance of proteins associated with either p190^BCR-ABL^ or p210^BCR-ABL^ Hantschel and coworkers used stable isotope labeling with amino acids in tissue culture or SILAC, combined with mass spectrometry [45]. This approach led to the identification of 147 BCR-ABL-associated proteins in BaF3 pro-B cells, 30 of which interacted specifically with p210^BCR-ABL^, 59 interacted specifically with p190^BCR-ABL,^ and 58 interacted with both [46]. STS-1, ShcA, and Cbl were all found to co-purify with both BCR-ABL proteins [46]. Interestingly, more STS-1 co-purified with p210^BCR-ABL^ [46,47]. The preference of STS-1 for association with p210^BCR-ABL^ was confirmed using co-immunoprecipitation experiments [46]. This preference may explain the observation that under specific conditions expression of p190^BCR-ABL^ results in a more aggressive form of leukemia when compared to expression of p210^BCR-ABL^ [48].

Co-immunoprecipitation experiments confirmed the interaction between p190^BCR-ABL^ and STS-1. Analysis of BCR-ABL1 deletion mutants showed that co-immunoprecipitation with STS-1 depends on the ABL part of p190^BCR-ABL^ [49]. Analysis of STS-1 mutants showed that neither a functional UBA, nor an SH3, nor a phosphatase domain was required for co-immunoprecipitation with p190^BCR-ABL^ [49]. It remains unclear exactly how BCR-ABL1 and STS-1 interact.

Expression of STS-1 reduced p190^BCR-ABL^ tyrosine phosphorylation. In addition, STS-1 reduces the rate of p190^BCR-ABL^-dependent cell proliferation in vitro and increases survival rates in mice injected with wild-type or STS-1/STS-2 deficient bone marrow cells expressing p210^BCR-ABL^. Thus STS-1 acts as an inhibitor of BCR-ABL1 transformation. Interestingly, robust tyrosine phosphorylation of STS-1 can be observed in HEK cells but only after inactivation of the STS-1 PGM domain. This suggests that BCR-ABL1 can phosphorylate STS-1 and that STS-1 can dephosphorylate itself [49]. It may be worth further investigating STS-1 tyrosine phosphorylation using the phosphatase-deficient mutant.

## 8. ShcA

The ShcA gene encodes three proteins of 46, 52, and 66 kDa that differ from each other only in the length of the amino-terminal region [50]. All three ShcA proteins are composed of an amino-terminal region, a PTB (phosphotyrosine-binding) domain, a central region containing several tyrosine phosphorylation sites, and a carboxy-terminal SH2 domain. PTB and SH2 domains bind to phosphorylated tyrosine residues in other proteins [50]. ShcA is best known for its role in signal transduction by receptor protein–tyrosine kinases [50]. Ligand binding to the receptor results in kinase activation and autophosphorylation, thereby creating one or more binding sites for ShcA. Following its association with the receptor, ShcA itself becomes phosphorylated on several tyrosine residues, thereby creating binding sites for other phosphotyrosine-binding proteins [51]. The principal binding partner for ShcA tyrosine phosphorylation sites is Grb2 [52]. Grb2 is a small adaptor protein that acts in concert with Sos to link protein–tyrosine kinases to activation of the Ras-MAP kinase pathway [52].

To identify novel ShcA binding proteins, we used a synthetic phosphopeptide, based on the ShcA tyrosine 317 phosphorylation site as an affinity reagent. Immobilized P.Tyr 317 peptides were incubated with lysates from A549 human carcinoma cells and bound proteins were resolved by SDS-PAGE and visualized by silver staining. After scaling up the experiment we identified a 72 kDa by mass spectrometry as STS-1 [53]. The identification and association with ShcA were confirmed by immunoblotting.

To study the interaction between STS-1 and ShcA in the context of protein–tyrosine kinase activation, COS-1 cells were stimulated with EGF for various amounts of time. ShcA immunoprecipitations were probed for the presence of STS-1. Our results show that STS-1 is associated with ShcA in unstimulated cells and that binding increases during the first two minutes of stimulation, after which it starts to go down [53]. Analysis of STS-1 shows that its levels are highest in unstimulated cells and that STS-1 protein levels start to decrease immediately upon stimulation [53]. The transient increase in the association between STS-1 and ShcA appears to result from an increase in binding sites and a decrease in STS-1 protein levels. The observation that STS-1 disappears when receptors are activated suggests that STS-1 could function in unstimulated cells, perhaps by keeping receptors in their inactive state in the absence of relevant amounts of ligand. Because STS-1 disappears upon activation of the EGF receptor in COS-1 cells, the possibility exists that it is targeted for degradation together with associated proteins.

## 9. Dynamin

Dynamins are cytosolic proteins that associate transiently with membranes where they are involved in the pinching of vesicles [54,55]. This is an important part of endocytosis and plays a pivotal role in intracellular vesicle trafficking. Dynamins contain multiple domains including a GTPase domain, a GTPase effector domain, a PH (pleckstrin homology) domain, and a Pro-rich region. The GTPase and GTPase effector domains are involved in vesicle formation [54,55]. The PH domain and the Pro-rich region are involved in membrane binding and association with other proteins [56,57]. PH domains act by binding to phosphoinositides [58].

Madshus and coworkers confirmed observations showing that overexpression of STS-1 or STS-2 reduces internalization of the EGF receptor [7,8]. To extend these observations, they investigated the effect of STS-2 overexpression on the uptake of transferrin and low-density lipoproteins and found uptake to be reduced when compared to control cells [59]. Further investigation showed that STS-2 overexpression also reduced the internalization of CD59 and MHC-I, two other proteins that are localized to the cell surface [59]. Internalization of transferrin, low-density lipoprotein, CD59, and MHC-1 requires the formation of endocytic vesicles and is dependent on the participation of dynamin [54,55]. Based on these observations they asked whether dynamin associates with STS-2. Immunofluorescence staining was used to show some colocalization of STS-2 and dynamin in cells [59]. Furthermore, HA-tagged dynamin was shown to co-immunoprecipitate with Myc-tagged STS-2. Co-immunoprecipitation was dependent on the presence of a functional STS-2 SH3 domain. Based on their observations Madshus and coworkers hypothesized that STS-2 uses its SH3 domain to bind to dynamin and that the STS-2 protein inhibits endocytosis by preventing dynamin from mobilizing to sites of vesicle formation. However, proof that STS-2 and dynamin interact directly was not provided in their report [59]. Thus, it remains possible that STS-2 uses its SH3 domain to bind to an unidentified intermediate that is associated with dynamin. If their model holds true, it suggests that STS-2 could interfere with various dynamin-dependent transport processes in the cell.

## 10. Apoptosis Inducing Factor

AIF (Apoptosis-inducing factor) was discovered as a mitochondrial protein that can induce various aspects of apoptosis in nuclei [60]. AIF is present in the mitochondrial intermembrane space and is loosely associated with the inner mitochondrial membrane, where it supports proper functioning of the mitochondrial electron transport chain [61]. It is thought that certain apoptotic stimuli cause cleavage of AIF and its release from the inner mitochondrial membrane, after which it moves from mitochondria through the cytoplasm into the nucleus, where it contributes to the onset of apoptosis [62]. Tsygankov, Carpino, and coworkers purified Flag-tagged STS-2 using anti-Flag agarose from 293T cells and identified AIF as an associated protein by two-dimensional mass spectroscopy [63]. The association was confirmed by co-immunoprecipitation after overexpression of both STS-2 and AIF in 293T cells. Analysis of several STS-2 mutants suggests that AIF associates with the STS-2 amino terminus [63]. However, the SH3 domain was found to be dispensable for association with AIF [63]. Experiments in Jurkat T cells, in which STS-2 levels were experimentally reduced, show a diminished apoptotic response to serum deprivation. It remains unresolved whether the association between STS-2 and AIF is either direct or indirect. These observations suggest that STS-2 could be involved in the regulation or execution of the apoptotic response.

## 11. Conclusions

The STS-1 and STS-2 proteins are involved in the regulation of protein–tyrosine kinases. They use their SH3 domain to bind to proteins that are thought to be involved in stimulating endocytosis and degradation of activated protein–tyrosine kinases, including Cbl, CIN85, and dynamin (Table 1). Most of the studies support the idea that STS-1 and STS-2 inhibit the targeting of active protein–tyrosine kinases for degradation. In the case of STS-1, this is likely the result of dephosphorylation of the protein–tyrosine kinases, thereby preventing negative regulators from binding. This is consistent with the observation that the PGM domain is required for STS-1-dependent inhibition of receptor degradation. Kinase dephosphorylation is likely to reduce association with downstream mediators of signaling. Thus STS-1 appears to inhibit both protein–tyrosine kinase downregulation as well as signaling.

STS-2, which lacks robust protein phosphatase activity, could use its SH3 domain to sequester negative regulators and prevent them from interacting with active protein–tyrosine kinases. It may even be possible that STS-2 targets some of these negative regulators for degradation. This is likely to result in prolonged protein–tyrosine kinase signaling. While receptor stabilization by STS-2 was shown to require a functional UBA domain in several studies, it remains unclear exactly how the UBA domain contributes to this process. It is possible that the STS-2 UBA domain binds to ubiquitinated receptors thereby preventing other ubiquitin-binding proteins from targeting those receptors for internalization or degradation.

Exactly how STS-1 or STS-2 recognize activated protein–tyrosine kinase remains unresolved. This holds true for the interactions with ShcA and AIF as well. Interestingly, these interactions most often involve docking onto tyrosine phosphorylated binding sites. Neither STS-1 nor STS-2 contains a domain that is known to bind in a sequence-specific manner to phosphotyrosine. This suggests that the interaction with these tyrosine phosphorylated proteins may be indirect. Candidate proteins that could mediate these phosphotyrosine-dependent interactions are Cbl and ShcA.

The interactions between STS-1 and STS-2 and Cbl are well documented. STS-2 has been shown to use its SH3 domain to bind to the proline-rich region in Cbl. This leaves the Cbl TKB domain to mediate the interaction with tyrosine phosphorylated receptors. However, this model is inconsistent with some of the data showing that the binding of STS-1 to the EGF receptor is independent of Cbl. It remains possible that protein–tyrosine kinase recognition is different for STS-1 as compared to STS-2.

A second candidate protein that could mediate the interaction between STS-1 or STS-2 and active protein–tyrosine kinases is ShcA. ShcA has been identified in several studies as a binding partner for STS-1. ShcA also happens to be well-equipped to function as an intermediate between STS-1 or STS-2 and their targets, because it carries a PTB domain as well as an SH2 domain. Both domains mediate phosphotyrosine-dependent protein–protein interactions. Unfortunately, the interaction between ShcA and STS-1 remains poorly characterized.

Currently, the function of the STS-1 PGM domain and the function of the STS-2 SH3 domain are reasonably well understood. In contrast, it remains unclear how the UBA domains, the esterase domains, and the STS-2 PGM domain contribute to the regulation of protein–tyrosine kinase signaling. Finally, finding out exactly how STS-1 and STS-2 engage active protein–tyrosine kinases is important and this warrants further investigation.

## Figures and Tables

**Table 1:** Protein–protein interactions involving STS-1 and STS-2.

Binding Partner	Family Member	Domain	References
JAK2	STS-1	not determined	[4]
Syk	STS-1	not determined	[22]
	STS-2	not determined	[22]
CIN85	STS-2	SH3, likely	[25]
Cbl	STS-2	SH3	[7,8]
EGF receptor	STS-1	not determined	[7,28]
BCR-ABL1	STS-1	not determined	[37,42]
ShcA	STS-1	not determined	[53]
Dynamin	STS-2	SH3	[59]
AIF	STS-2	not determined	[63]

## References

[1] Wattenhofer M., Shibuya K., Kudoh J., Lyle R., Michaud J., Rossier C., Kawasaki K., Asakawa S., Minoshima S., Berry A. (2001). Isolation and characterization of the UBASH3A gene on 21q22.3 encoding a potential nuclear protein with a novel combination of domains. Hum. Genet..

[2] Mazumder R., Iyer L.M., Vasudevan S., Aravind L. (2002). Detection of novel members, structure-function analysis and evolutionary classification of the 2H phosphoesterase superfamily. Nucleic Acids Res..

[3] Gadina M., Hilton D., Johnston J.A., Morinobu A., Lighvani A., Zhou Y.J., Visconti R., O’Shea J.J. (2001). Signaling by type I and II cytokine receptors: Ten years after. Curr. Opin. Immunol..

[4] Carpino N., Kobayashi R., Zang H., Takahashi Y., Jou S.T., Feng J., Nakajima H., Ihle J.N. (2002). Identification, cDNA cloning, and targeted deletion of p70, a novel, ubiquitously expressed SH3 domain-containing protein. Mol. Cell. Biol..

[5] Rao N., Dodge I., Band H. (2002). The Cbl family of ubiquitin ligases: Critical negative regulators of tyrosine kinase signaling in the immune system. J. Leukoc. Biol..

[6] Thien C.B., Langdon W.Y. (2005). c-Cbl and Cbl-b ubiquitin ligases: Substrate diversity and the negative regulation of signalling responses. Biochem. J..

[7] Feshchenko E.A., Smirnova E.V., Swaminathan G., Teckchandani A.M., Agrawal R., Band H., Zhang X., Annan R.S., Carr S.A., Tsygankov A.Y. (2004). TULA: An SH3- and UBA-containing protein that binds to c-Cbl and ubiquitin. Oncogene.

[8] Kowanetz K., Crosetto N., Haglund K., Schmidt M.H., Heldin C.H., Dikic I. (2004). Suppressors of T-cell receptor signaling Sts-1 and Sts-2 bind to Cbl and inhibit endocytosis of receptor tyrosine kinases. J. Biol. Chem..

[9] Dikic I., Wakatsuki S., Walters K.J. (2009). Ubiquitin-binding domains—From structures to functions. Nat. Rev. Mol. Cell Biol..

[10] Mayer B.J. (2001). SH3 domains: Complexity in moderation. J. Cell Sci..

[11] Jedrzejas M.J. (2000). Structure, function, and evolution of phosphoglycerate mutases: Comparison with fructose-2,6-bisphosphatase, acid phosphatase, and alkaline phosphatase. Prog. Biophys. Mol. Biol..

[12] Mikhailik A., Ford B., Keller J., Chen Y., Nassar N., Carpino N. (2007). A phosphatase activity of Sts-1 contributes to the suppression of TCR signaling. Mol. Cell.

[13] Chen X., Ren L., Kim S., Carpino N., Daniel J.L., Kunapuli S.P., Tsygankov A.Y., Pei D. (2010). Determination of the substrate specificity of protein-tyrosine phosphatase TULA-2 and identification of Syk as a TULA-2 substrate. J. Biol. Chem..

[14] San Luis B., Sondgeroth B., Nassar N., Carpino N. (2011). Sts-2 is a phosphatase that negatively regulates zeta-associated protein (ZAP)-70 and T cell receptor signaling pathways. J. Biol. Chem..

[15] Hu X., Li J., Fu M., Zhao X., Wang W. (2021). The JAK/STAT signaling pathway: From bench to clinic. Signal Transduct. Target. Ther..

[16] Hubbard S.R. (2017). Mechanistic Insights into Regulation of JAK2 Tyrosine Kinase. Front. Endocrinol..

[17] Pawson T. (1995). Protein modules and signalling networks. Nature.

[18] Latour S., Veillette A. (2001). Proximal protein tyrosine kinases in immunoreceptor signaling. Curr. Opin. Immunol..

[19] Wang H., Kadlecek T.A., Au-Yeung B.B., Goodfellow H.E., Hsu L.Y., Freedman T.S., Weiss A. (2010). ZAP-70: An essential kinase in T-cell signaling. Cold Spring Harb. Perspect. Biol..

[20] Carpino N., Turner S., Mekala D., Takahashi Y., Zang H., Geiger T.L., Doherty P., Ihle J.N. (2004). Regulation of ZAP-70 activation and TCR signaling by two related proteins, Sts-1 and Sts-2. Immunity.

[21] Carpino N., Chen Y., Nassar N., Oh H.-W. (2009). The Sts proteins target tyrosine phosphorylated, ubiquitinated proteins within TCR signaling pathways. Mol. Immunol..

[22] Agrawal R., Carpino N., Tsygankov A. (2008). TULA proteins regulate activity of the protein tyrosine kinase Syk. J. Cell. Biochem..

[23] Havrylov S., Redowicz M.J., Buchman V.L. (2010). Emerging roles of Ruk/CIN85 in vesicle-mediated transport, adhesion, migration and malignancy. Traffic.

[24] Dikic I. (2002). CIN85/CMS family of adaptor molecules. FEBS Lett..

[25] Kong M.S., Hashimoto-Tane A., Kawashima Y., Sakuma M., Yokosuka T., Kometani K., Onishi R., Carpino N., Ohara O., Kurosaki T. (2019). Inhibition of T cell activation and function by the adaptor protein CIN85. Sci. Signal.

[26] Marmor M.D., Yarden Y. (2004). Role of protein ubiquitylation in regulating endocytosis of receptor tyrosine kinases. Oncogene.

[27] Thien C.B., Langdon W.Y. (2001). Cbl: Many adaptations to regulate protein tyrosine kinases. Nat. Rev. Mol. Cell Biol..

[28] Raguz J., Wagner S., Dikic I., Hoeller D. (2007). Suppressor of T-cell receptor signalling 1 and 2 differentially regulate endocytosis and signalling of receptor tyrosine kinases. FEBS Lett..

[29] Cilloni D., Saglio G. (2012). Molecular pathways: BCR-ABL. Clin. Cancer Res..

[30] Melo J.V., Barnes D.J. (2007). Chronic myeloid leukaemia as a model of disease evolution in human cancer. Nat. Rev. Cancer.

[31] Wu P., Nielsen T.E., Clausen M.H. (2015). FDA-approved small-molecule kinase inhibitors. Trends Pharmacol. Sci..

[32] Peiris M.N., Li F., Donoghue D.J. (2019). BCR: A promiscuous fusion partner in hematopoietic disorders. Oncotarget.

[33] Bos J.L., Rehmann H., Wittinghofer A. (2007). GEFs and GAPs: Critical elements in the control of small G proteins. Cell.

[34] Wang J.Y. (2014). The capable ABL: What is its biological function?. Mol. Cell. Biol..

[35] Butturini A., Arlinghaus R.B., Gale R.P. (1996). BCR/ABL and leukemia. Leuk. Res..

[36] Lozzio C.B., Lozzio B.B. (1975). Human chronic myelogenous leukemia cell-line with positive Philadelphia chromosome. Blood.

[37] Brehme M., Hantschel O., Colinge J., Kaupe I., Planyavsky M., Kocher T., Mechtler K., Bennett K.L., Superti-Furga G. (2009). Charting the molecular network of the drug target Bcr-Abl. Proc. Natl. Acad. Sci. USA.

[38] Burckstummer T., Bennett K.L., Preradovic A., Schutze G., Hantschel O., Superti-Furga G., Bauch A. (2006). An efficient tandem affinity purification procedure for interaction proteomics in mammalian cells. Nat. Methods.

[39] Kirchhausen T. (1999). Adaptors for clathrin-mediated traffic. Annu. Rev. Cell Dev. Biol..

[40] Chapman-Smith A., Cronan J.E. (1999). The enzymatic biotinylation of proteins: A post-translational modification of exceptional specificity. Trends Biochem. Sci..

[41] Roux K.J., Kim D.I., Raida M., Burke B. (2012). A promiscuous biotin ligase fusion protein identifies proximal and interacting proteins in mammalian cells. J. Cell Biol..

[42] Cutler J.A., Udainiya S., Madugundu A.K., Renuse S., Xu Y., Jung J., Kim K.P., Wu X., Pandey A. (2020). Integrative phosphoproteome and interactome analysis of the role of Ubash3b in BCR-ABL signaling. Leukemia.

[43] Braiman A., Isakov N. (2015). The Role of Crk Adaptor Proteins in T-Cell Adhesion and Migration. Front. Immunol..

[44] Thomas M.P., Erneux C., Potter B.V. (2017). SHIP2: Structure, Function and Inhibition. ChemBioChem.

[45] Oda Y., Huang K., Cross F.R., Cowburn D., Chait B.T. (1999). Accurate quantitation of protein expression and site-specific phosphorylation. Proc. Natl. Acad. Sci. USA.

[46] Reckel S., Hamelin R., Georgeon S., Armand F., Jolliet Q., Chiappe D., Moniatte M., Hantschel O. (2017). Differential signaling networks of Bcr-Abl p210 and p190 kinases in leukemia cells defined by functional proteomics. Leukemia.

[47] Cutler J.A., Tahir R., Sreenivasamurthy S.K., Mitchell C., Renuse S., Nirujogi R.S., Patil A.H., Heydarian M., Wong X., Wu X. (2017). Differential signaling through p190 and p210 BCR-ABL fusion proteins revealed by interactome and phosphoproteome analysis. Leukemia.

[48] Li S., Ilaria R.L., Million R.P., Daley G.Q., Van Etten R.A. (1999). The P190, P210, and P230 forms of the BCR/ABL oncogene induce a similar chronic myeloid leukemia-like syndrome in mice but have different lymphoid leukemogenic activity. J. Exp. Med..

[49] Mian A.A., Baumann I., Liebermann M., Grebien F., Superti-Furga G., Ruthardt M., Ottmann O.G., Hantschel O. (2019). The phosphatase UBASH3B/Sts-1 is a negative regulator of Bcr-Abl kinase activity and leukemogenesis. Leukemia.

[50] Ravichandran K.S. (2001). Signaling via Shc family adapter proteins. Oncogene.

[51] van der Geer P., Wiley S., Gish G.D., Pawson T. (1996). The Shc adaptor protein is highly phosphorylated at conserved, twin tyrosine residues (Y239/240) that mediate protein-protein interactions. Curr. Biol..

[52] Downward J. (1994). The GRB2/Sem-5 adaptor protein. FEBS Lett..

[53] van der Meulen T., Swarts S., Fischer W., van der Geer P. (2017). Identification of STS-1 as a novel ShcA-binding protein. Biochem. Biophys. Res. Commun..

[54] Antonny B., Burd C., De Camilli P., Chen E., Daumke O., Faelber K., Ford M., Frolov V.A., Frost A., Hinshaw J.E. (2016). Membrane fission by dynamin: What we know and what we need to know. EMBO J..

[55] Gonzalez-Jamett A.M., Momboisse F., Haro-Acuna V., Bevilacqua J.A., Caviedes P., Cardenas A.M. (2013). Dynamin-2 function and dysfunction along the secretory pathway. Front. Endocrinol..

[56] Shpetner H.S., Herskovits J.S., Vallee R.B. (1996). A binding site for SH3 domains targets dynamin to coated pits. J. Biol. Chem..

[57] Lee A., Frank D.W., Marks M.S., Lemmon M.A. (1999). Dominant-negative inhibition of receptor-mediated endocytosis by a dynamin-1 mutant with a defective pleckstrin homology domain. Curr. Biol..

[58] Lemmon M.A., Ferguson K.M., Abrams C.S. (2002). Pleckstrin homology domains and the cytoskeleton. FEBS Lett..

[59] Bertelsen V., Breen K., Sandvig K., Stang E., Madshus I.H. (2007). The Cbl-interacting protein TULA inhibits dynamin-dependent endocytosis. Exp. Cell Res..

[60] Susin S.A., Lorenzo H.K., Zamzami N., Marzo I., Snow B.E., Brothers G.M., Mangion J., Jacotot E., Costantini P., Loeffler M. (1999). Molecular characterization of mitochondrial apoptosis-inducing factor. Nature.

[61] Vahsen N., Cande C., Briere J.J., Benit P., Joza N., Larochette N., Mastroberardino P.G., Pequignot M.O., Casares N., Lazar V. (2004). AIF deficiency compromises oxidative phosphorylation. EMBO J..

[62] Otera H., Ohsakaya S., Nagaura Z., Ishihara N., Mihara K. (2005). Export of mitochondrial AIF in response to proapoptotic stimuli depends on processing at the intermembrane space. EMBO J..

[63] Collingwood T.S., Smirnova E.V., Bogush M., Carpino N., Annan R.S., Tsygankov A.Y. (2007). T-cell ubiquitin ligand affects cell death through a functional interaction with apoptosis-inducing factor, a key factor of caspase-independent apoptosis. J. Biol. Chem..

